# Genome Sequence of *Pseudomonas* Phage UMP151, Isolated from the Female Bladder Microbiota

**DOI:** 10.1128/MRA.00853-19

**Published:** 2019-08-15

**Authors:** Genevieve Johnson, Catherine Putonti

**Affiliations:** a Bioinformatics Program, Loyola University Chicago, Chicago, Illinois, USA; b Department of Biology, Loyola University Chicago, Chicago, Illinois, USA; c Department of Computer Science, Loyola University Chicago, Chicago, Illinois, USA; d Department of Microbiology and Immunology, Loyola University Chicago, Maywood, Illinois, USA; DOE Joint Genome Institute

## Abstract

A temperate bacteriophage, designated UMP151, was isolated from a Pseudomonas aeruginosa strain from a catheterized urine sample of a woman with overactive bladder (OAB) symptoms. The 41,303-bp genome sequence of *Pseudomonas* phage UMP151 exhibits sequence similarity to prophage and lytic phage sequences isolated from other areas of the human body.

## ANNOUNCEMENT

Prior studies of the female urinary microbiota have identified associations between bladder bacteria and urinary symptoms (1–6). Recent surveys have found that viruses, particularly bacteriophages (phages), far outnumber bacteria in the urinary tract (7) and that the majority of these bacterial members are lysogens, some harboring upwards of 10 intact prophages (8). In our ongoing effort to characterize the phage population of the urinary tract microbiota, we describe here the genome sequence of a new isolate, *Pseudomonas* phage UMP151, a temperate phage induced from a Pseudomonas aeruginosa strain isolated from the urine collected via transurethral catheterization from a woman with overactive bladder (OAB) symptoms. P. aeruginosa is not frequently found within the bladder microbiota of healthy women (9); rather, it is an opportunistic pathogen of the urinary tract typically associated with nosocomial urinary tract infections (10). Furthermore, no association between P. aeruginosa and OAB has been identified (9). *Pseudomonas* phage UMP151 is the fourth *Pseudomonas* phage isolated from the urinary tract to be described in the literature (11–13) and the first genome of a *Pseudomonas* phage from the bladder microbiota.

P. aeruginosa strain UMB0151 was isolated via culture from a prior study (1, 2, 6, 9) and stored at −80°C. We streaked this bacterial strain on an LB agar plate and grew it overnight at 37°C. A single colony was selected to inoculate liquid LB medium and was grown overnight with shaking at 37°C. The overnight culture was centrifuged for 8 min at 13,000 × *g* and filtered through a 0.2-μm cellulose acetate syringe filter, and then the filtrate was spotted (10 μl) onto a lawn of P. aeruginosa ATCC 15692. The plate was incubated overnight at 37°C. Plaques were found where the filtrate was spotted. Spots were harvested, suspended in LB medium, and filtered using a 0.2-μm cellulose acetate syringe filter. A sample of 300 μl was pipetted and treated with 5 U of Optizyme DNase I (Fisher BioReagents) prior to DNA extraction using the Quick-DNA kit (Zymo) following the manufacturer’s instructions. The Nextera XT DNA library preparation kit was used, and the library was sequenced on an Illumina MiSeq sequencer using the MiSeq reagent kit v2 (500 cycles), producing 278,404 paired-end 250-bp reads. Raw reads were first trimmed for quality using sickle (https://github.com/najoshi/sickle) and then assembled by SPAdes (v3.11.1) (14) using the “only assembler” option for *k* value of 55, 77, 99, and 127. In total, 4,828 assembled contigs were produced, ranging in size from 128 to 41,303 bp. Coverage was calculated using BBmap v38.47 (http://sourceforge.net/projects/bbmap). The largest contig, representative of the complete genome sequence of UMP151, had a significantly greater coverage (768×) than the other contigs assembled (∼1×). The genome termini were determined using PhageTerm v1.0.12 (15) via Galaxy (16), and the genome was annotated via RAST using the Classic RAST pipeline (17).

The complete genome for *Pseudomonas* phage UMP151 is 41,303 bp with a GC content of 63.2%. The genome encodes 56 genes. The complete genome sequence was queried against the NCBI nr/nt database via MegaBLAST, identifying 100% sequence identity and coverage with P. aeruginosa strain MRSN12280 (GenBank accession number CP028162), which was isolated from the sacrum of an individual. The best hit to an isolated phage was to the siphovirus *Pseudomonas* phage CF5 (GenBank accession number MK511057; query coverage, 77%; sequence identity, 97.48%), which was isolated from the lung of a cystic fibrosis patient (18).

### Data availability.

The draft whole-genome project for *Pseudomonas* phage UMP151 has been deposited at DDBJ/EMBL/GenBank under accession number MK934841. Raw sequence reads are deposited at DDBJ/EMBL/GenBank under accession number SRR9072121, which is part of BioProject number PRJNA494532.
